# Organotellurium-mediated living radical polymerization under photoirradiation by a low-intensity light-emitting diode

**DOI:** 10.3762/bjoc.9.183

**Published:** 2013-08-07

**Authors:** Yasuyuki Nakamura, Shigeru Yamago

**Affiliations:** 1 Institute for Chemical Research, Kyoto University, Gokasyo, Uji, Kyoto 611-0011, Japan, and Core Research for Evolutional Science and Technology (CREST), Japan Science and Technology Agency (JST)

**Keywords:** free radical, light-emitting diode, living radical polymerization, organotellurium compound, photopolymerization, tellurium, visible light

## Abstract

A low-intensity (6 W) light-emitting diode (LED) effectively activated an organotellurium chain transfer agent and the dormant species, promoting well-controlled radical polymerization. The use of the LED provided many advantages over the previously reported high-intensity Hg lamp, including high energy efficiency during the polymerization, and easy availability of the low-cost light source. Structurally well-defined poly(methyl methacrylate), poly(methyl acrylate), and polystyrene, with narrow molecular weight distributions, were synthesized under LED irradiation with or without a neutral density filter.

## Introduction

Living radical polymerization (LRP) is one of the most powerful methods for the synthesis of structurally well-defined polymers because of its robustness and high versatility, which allows for the polymerization of a wide variety of vinyl monomers with various functionalities [1–3]. LRP relies on the reversible generation of polymer-end radicals from a dormant species. One approach to the activation of the dormant species is the use of photostimulation. This has been widely employed in conventional radical polymerization for various applications such as coatings, adhesives, gels and microelectronics [4–7]. The major motivation for the utilization of photochemistry in LRP is that it enables the dormant species to be activated under mild thermal conditions [8–11]. In addition, photochemical activation is beneficial for increasing the fidelity of the polymer-end structure [12]. However, the experimental setup required for the reaction provides problems, as distinctive light sources such as γ-rays or high-intensity UV irradiation are required [13–21].

**Figure 1:**
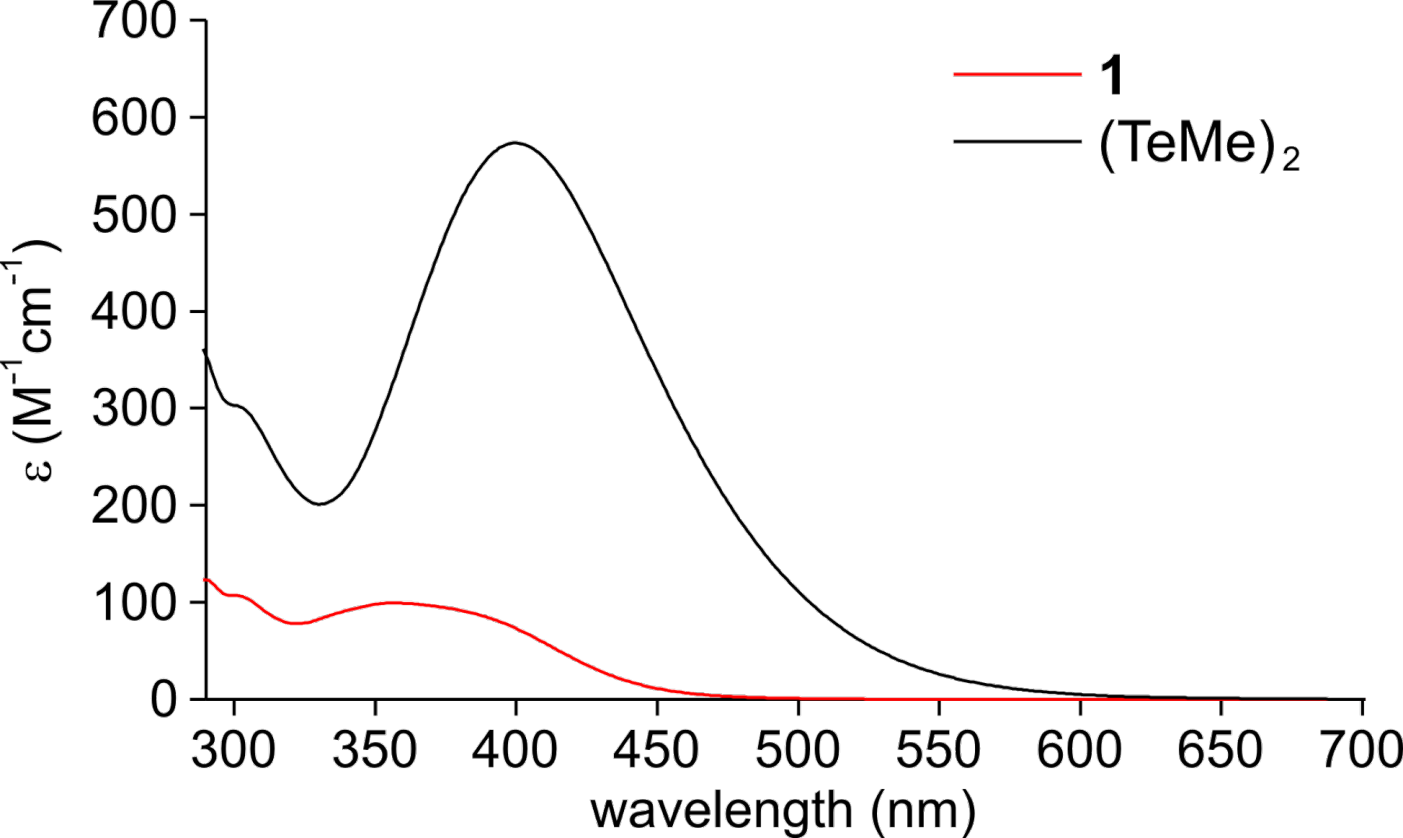
UV–vis absorption spectra of organotellurium chain transfer agent **1** and dimethyl ditelluride in toluene.

**Scheme 1:**
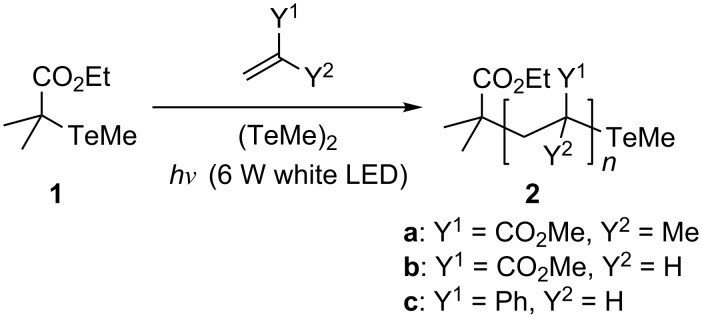
Photopolymerization in the presence of organotellurium chain transfer agent **1**.

We have previously developed organotellurium-mediated LRP (TERP), which has several synthetic advantages over other LRP methods [22–24]. These include high monomer versatility [25–27], good compatibility with polar functional groups and solvents [28–29], and facile living-end transformation for the synthesis of block copolymers [30–34] and end-functional polymers [35–36]. Furthermore, we recently reported that photochemical stimuli were efficient in the activation of organotellurium dormant species, and that TERP proceeded under mild thermal conditions to give highly controlled polymers [37]. The polymerization proceeded by irradiation with a weak-intensity light source such as a 60–100 W black lamp or sunlight, but we routinely used a high-intensity light source, namely, a 500 W high pressure Hg lamp, combined with a light cutoff filter. However, control of the light intensity was difficult under such conditions. For example, when light of a relatively high intensity was used to prepare a polyisoprene using TERP, a radical coupling reaction occurred because of the efficient formation of the polymer-end radical [38]. In addition, when ditellurides were added to the TERP reaction in order to increase the level of control over the polymerization of methacrylates, optimization of the conditions was difficult, because ditellurides have a stronger absorption coefficient than the organotellurium compounds (Figure 1). This resulted in the generation of tellurium radicals, which activate the organotellurium dormant species [30,39]. Therefore, the development of new photochemical conditions which employ a weak-intensity, readily available light source is necessary for expanding the utility of photo-TERP. We focused on a light emitting diode (LED) due to its high power conversion efficiency, low heat generation, narrow and tunable wavelength range of the emitted light, and ease of availability. Herein, we report on the use of photo-TERP with a 6 W white, household LED (Scheme 1). Attention was focused on the TERP of methyl methacrylate (MMA) in the presence of ditelluride, but other typical conjugated monomers, namely, methyl acrylate and styrene, were also evaluated. TERP of these monomers proceeded efficiently in a controlled manner by adjusting the light intensity using ND filters. The results clearly demonstrate the extremely high sensitivity of organotellurium compounds for generating radical species by photostimulation. Based on these findings, we also adjust our previous statements regarding the effect of ditelluride on TERP.

## Results and Discussion

The polymerization of MMA (100 equiv) was first conducted in the presence of organotellurium chain transfer agent **1** (1 equiv) and dimethyl ditelluride (1 equiv) under 6 W white LED at 70 °C (Scheme 1 and Table 1, run 1). The polymerization progressed rapidly, reaching 93% monomer conversion after 1 h. However, the number-average molecular weight (*M*_n_) of the resulting polymer (**2a**, *M*_n(exp)_ = 11900) deviated slightly from the theoretical value, as calculated from the monomer/**1** ratio and the monomer conversion (*M*_n(theo)_ = 9400). In addition, the control of molecular weight distribution (MWD, *M*_w_/*M*_n_ = 1.26; *M*_w_ refers to weight average molecular weight) was moderate.

**Table 1:** Photopolymerization of MMA in the presence of **1** under LED irradiation.^a^

Run	MMA/**1** ratio	ND filter^b^	Time (h)	Conv. (%)^c^	*M*_n(theo)_	*M*_n(exp)_^d^	*M*_w_/*M*_n_^d^

1	100	none	1.0	93	9400	11900	1.26
2	100	50	2.0	92	9300	10700	1.20
3	100	30	2.5	93	9400	9800	1.19
4	100	20	4.0	92	9300	9500	1.18
5	100	10	5.5	93	9500	10400	1.18
6^e^	100	none	12	36	3700	2500	1.27
7^f^	100	20	6.0	94	9500	10400	1.18
8^g^	100	20	3.0	91	9200	9600	1.19
9^h^	100	20	2.5	93	9400	9900	1.19
10	200	20	4.0	92	18500	20500	1.17
11	300	20	4.5	94	28400	31900	1.14
12^i^	500	10	5.0	94	47200	52400	1.14
13^i^	1000	10	5.5	91	91200	109700	1.25

^a^A solution of **1**, dimethyl ditelluride (1 equiv) and monomer (100 equiv) was irradiated with a 6 W LED with or without a ND filter at 70 °C. ^b^% Transmittance is shown. ^c^Determined by ^1^H NMR. ^d^Determined by GPC calibrated with PMMA standards. ^e^The reaction was carried out in the dark. ^f^The polymerization was carried out at 60 °C. ^g^The polymerization was carried out at 80 °C. ^h^The polymerization was carried out at 90 °C. ^i^2 equiv of dimethyl ditelluride was used.

**Figure 2:**
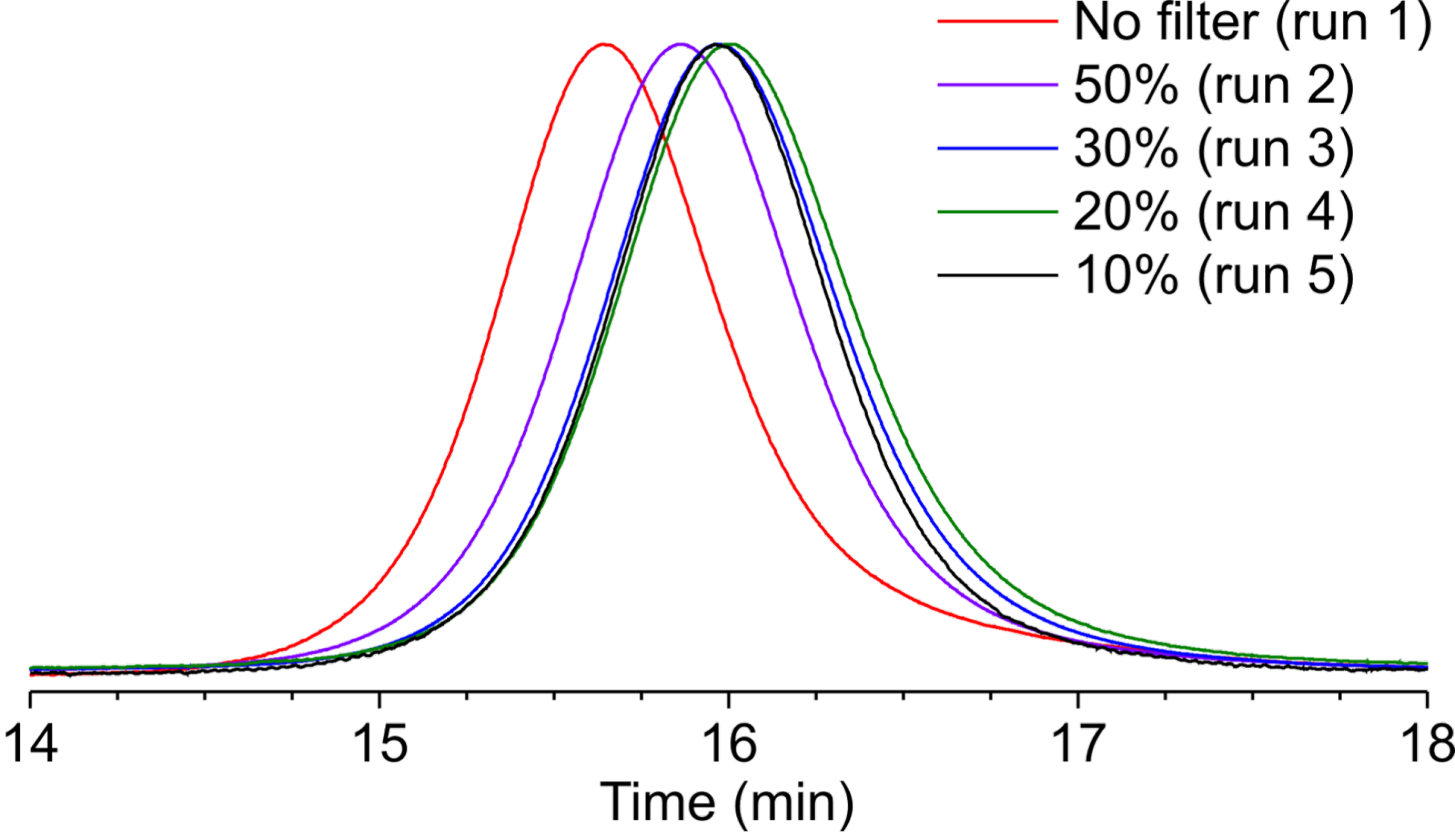
GPC traces for the polymerizations of MMA (Table 1). The percentages in the legend refer to the ND filters used.

We have previously reported that the intensity of irradiating light is critical for gaining control of the polymerization [37]. Therefore, we investigated the effect of the light intensity by using ND filters (Figure 2). When a 50% transmittance ND filter was used, the MWD improved to 1.20 from 1.26, while 2 h was required to reach >90% monomer conversion (Table 1, run 2). The MWD control was further improved when the light intensity was reduced by using 30% and 20% transmittance ND filters (*M*_w_/*M*_n_ = 1.18–1.19), although the monomer conversion further slowed down with this order (Table 1, runs 3 and 4). The MWD control reached a plateau when a 10% transmittance ND filter was used, while the polymerization rate was further decelerated (Table 1, run 5). In the absence of light irradiation, the polymerization was extremely slow, reaching only 36% monomer conversion, even after 12 h (Table 1, run 6). These results clearly demonstrate that weak light intensity was sufficient for activating the organotellurium compounds. The concentration of the radical species was roughly proportional to the light intensity. The monomer conversion after 1 h increased nearly linearly by the use of ND filters with higher transmittance (25, 36, 47, 62, and 93% monomer conversion by using 10, 20, 30, 50% ND filter and direct irradiation, respectively). The poor MWD control under high-intensity light is probably due to the increase in undesired termination reactions of the polymer-end radicals. When the termination is negligible, the level of MWD control is determined by the rate of deactivation of the polymer-end radicals by ditelluride forming a dormant species [39] which is independent of the light intensity.

The synthetic scope of MMA polymerization was next examined (Table 1, runs 7–13). As the generation of the polymer-end radical from the organotellurium dormant species does not require thermal stimuli, the reaction temperature can be arbitrarily selected depending on the propagation rate of the particular monomer. For example, the same polymerization proceeded over the range of 60–90 °C under otherwise identical conditions, affording structurally well controlled PMMAs in all cases (Table 1, runs 7–9). The conversion of the monomer was slow at lower temperatures because of the decrease in propagation rate, but the MWDs were identical in all cases, within the experimental error. Due to the high propagation rate at high temperatures the polymerization at 90 °C reached >90% monomer conversion within 2.5 h, affording PMMA with a narrow MWD (*M*_w_/*M*_n_ = 1.19, Table 1, run 9).

Structurally well controlled, high-molecular-weight PMMAs were also prepared by changing the monomer/**1** ratio (Table 1, runs 10–13). When 200–1000 equivalents of MMA over **1** were employed, monomer conversion reached >90% in all cases within 5.5 h, and PMMAs with a *M*_n,_ of 20500–109700 with a narrow MWD (*M*_w_/*M*_n_ ≤ 1.25) were synthesized. When more than 500 equivalents of monomer were employed, the addition of two equivalents of dimethyl ditelluride, as well as the use of a 10% transmittance ND filter, resulted in improved MWDs (Table 1, runs 12 and 13).

The use of the LED was also found to be effective for the efficient and controlled polymerization of other monomers (Table 2). For example, TERP of methyl acrylate (100 equiv) without ditelluride reached 91% monomer conversion after 1.6 h irradiation with the LED without an ND filter at 50 °C. Structurally well-defined poly(methyl acrylate) (PMA) with *M*_n_ = 10100 and a narrow MWD (*M*_w_/*M*_n_ = 1.11) was obtained (Table 2, run 1). High-molecular-weight PMAs with narrow MWDs (*M*_n_ = 90200, *M*_w_/*M*_n_ = 1.13 and *M*_n_ = 166000, *M*_w_/*M*_n_ = 1.15) were also prepared by changing the monomer/**1** ratio under LED irradiation through a 50% transmittance ND filter (Table 2, runs 2 and 3).

**Table 2:** Photopolymerization in the presence of **1** under LED irradiation.^a^

Run	Monomer (equiv)^b^	ND filter (% transmittance)	Temp. (°C)	Time (h)	Conv. (%)^c^	*M*_n(theo)_	*M*_n(exp)_^d^	*M*_w_/*M*_n_^d^

1	MA (100)	none	50	1.6	91	8000	10100	1.11
2	MA (1000)	50	50	5	93	80100	90200	1.13
3	MA (2000)	50	50	8	86	148100	166000	1.15
4	St (100)	none	90	6	96	10100	18200	1.36
5	St (100)	20	90	9	98	10300	11600	1.09
6	St (500)	10	90	14	95	49500	47400	1.18
7	St (1000)	10	90	16	83	86500	87500	1.33

^a^A solution of **1** and monomer (100 equiv) was irradiated with a 6 W LED with or without a ND filter. ^b^Monomer abbreviations: MA, methyl acrylate; St, styrene. ^c^Determined by ^1^H NMR. ^d^Determined by GPC calibrated with PMMA or polystyrene standards.

Next, the polymerization of styrene was examined at 90 °C, as the propagation rate constant of styrene is much lower than those of acrylates and methacrylates. Polymerization in the absence of a filter quantitatively converted the monomer to the polymer within 6 h (96%), but the *M*_n(exp)_ of the resulting polystyrene (18100) was significantly different from the *M*_n(theo)_ (10100), and the MWD control was unsatisfactory (*M*_w_/*M*_n_ = 1.36) (Table 2, run 4). On the other hand, when the polymerization was carried out through a 20% transmittance ND filter, although the monomer conversion was slower, the resulting polystyrene had a *M*_n(exp)_ close to *M*_n(theo)_, and a very narrow MWD (*M*_w_/*M*_n_ = 1.09) (Table 2, run 5). High-molecular-weight polystyrene of *M*_n_ = 47400 and 87500, with narrow MWDs (*M*_w_/*M*_n_ = 1.18 and 1.33, respectively) were successfully synthesized by using a 10% transmittance ND filter (Table 2, runs 6 and 7).

**Scheme 2:**
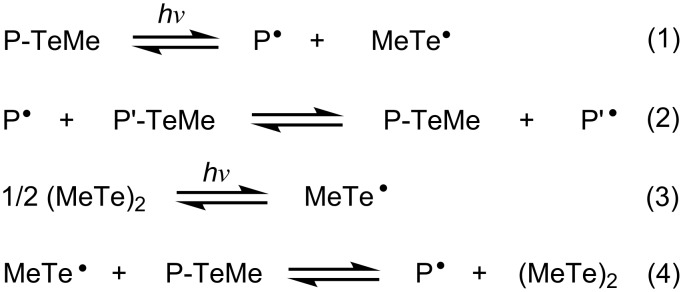
Activation and deactivation mechanism of dormant (P-TeMe) and ditelluride.

When the ditelluride is absent, polymerization is initiated by the direct photolysis of the carbon–tellurium bond of the organotellurium dormant species, P–TeMe (Scheme 2, reaction 1, P denotes polymer) [37]. Once the polymer radical is generated, it predominantly undergoes a degenerative chain transfer-mediated polymerization reaction (Scheme 2, reaction 2, P′ refers to a polymer with either the same or different chain length as P) [25,40]. When the ditelluride is present, its activation produces two molecules of methyltellanyl radical (Scheme 2, reaction 3). As ditelluride possesses a higher absorption coefficient in the UV region than organotellurium compounds such as **1** (Figure 1), the preferential activation of ditelluride over organotellurium compounds should occur. Once a methyltellanyl radical forms, it activates P–TeMe, giving a polymer end-radical and a ditelluride (Scheme 2, reaction 4).

We have previously reported that the addition of ditelluride accelerates the TERP and that the activation of P–TeMe by a methyltellanyl radical is the origin of this rate enhancement [39]. However, the mechanism by which this occurs has not been fully elucidated. As we have deduced that the activation of ditelluride successfully proceeds under very weak intensity light irradiation, the tellanyl radical should also be formed by the photons from interior fluorescent lighting. Indeed, TERP of MMA in the presence of ditelluride completed in approximately 13 h at 80 °C, without any special caution under interior fluorescent lighting [30], whereas the same experiment completely protected from all light sources took 22 h.

## Conclusion

A new and efficient procedure for photoinduced TERP was developed by using a low energy (6 W) visible LED as the light source. Compared to previously employed photoinduced TERP by using a 60–500 W light source, the energy efficiency was significantly improved. As the TERP of MMA and styrene was best controlled in combination with 10 or 20% transmittance ND filters, light with an intensity below 6 W should also be usable. Improvement in the MWD could be achieved by appropriate tuning of the light intensity. As activation of the organotellurium compounds has been shown to be possible with a low power and readily available LED, implementation to large scale synthesis should be feasible. Furthermore, the activation of the dormant species does not require thermal stimuli, and so independent control of the initiation (radical generation) and the propagation should be possible.

## Experimental

**General**: All reactions involving oxygen and moisture sensitive compounds were carried out in a dry reaction vessel under a nitrogen atmosphere. A 6 W white LED (Panasonic) was used as the light source in combination with a neutral density (ND) filter (Sigma Koki). ^1^H NMR (400 MHz) spectra were measured for a CDCl_3_ solution of the sample and are reported in ppm (δ) from an internal of tetramethylsilane. Gel permeation chromatography (GPC) was performed on a machine equipped with two linearly connected polystyrene mixed gel columns (Shodex LF-604) at 40 °C by using UV and refluctive index (RI) detectors with chloroform as the eluent. The number-average molecular weight (*M*_n_) is reported in g·mol^−1^. PMMA and poly(methyl acrylate) were calibrated with PMMA standards, and polystyrene was calibrated with polystyrene standards.

**Materials**: Unless otherwise noted, chemicals obtained from commercial suppliers were used as received. Methyl methacrylate (MMA), methyl acrylate and styrene were washed with 5% NaOH aqueous solution and were distilled over CaH_2_. Ethyl 2-methyltellanylisobutylate (**1**) and dimethyl ditelluride were prepared as reported [22]. The UV–vis spectra of **1** and dimethyl ditelluride are shown in Figure 1.

**Figure 3:**
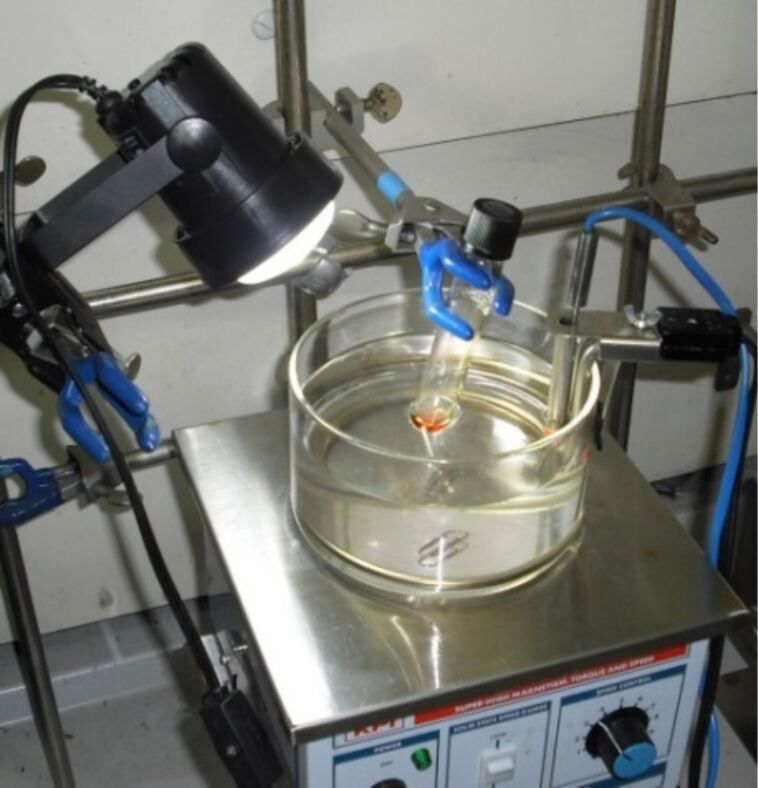
Experimental setup for the photopolymerization using the LED.

**Typical procedure for photopolymerization**: A solution of MMA (1.0 mL, 9.4 mmol), **1** (16.5 μL, 0.094 mmol), and dimethyl ditelluride (10.0 μL, 0.094 mmol) was irradiated with a 6 W white LED equipped with a 20% ND filter at 70 °C for 4 h under a nitrogen atmosphere in a capped tube (Figure 3). A small portion of the reaction mixture was then removed, and the conversion of the monomer (92%) was determined by using ^1^H NMR spectroscopy. The reaction mixture was analyzed by using GPC, and the *M*_n_ (9500) and *M*_w_/*M*_n_ (1.18) were determined. Chloroform was subsequently added to the mixture, and the resulting solution was poured into hexane with vigorous stirring. The product was collected by suction filtration and dried under vacuum to give the final PMMA product (810 mg).
